# Progress of Research in In Situ Smart Hydrogels for Local Antitumor Therapy: A Review

**DOI:** 10.3390/pharmaceutics14102028

**Published:** 2022-09-23

**Authors:** Juan Zhao, Ling Wang, Haiwei Zhang, Bin Liao, Yongsheng Li

**Affiliations:** Department of Phase I Clinical Trial Center, Chongqing University Cancer Hospital, Chongqing 400030, China

**Keywords:** in situ hydrogel, stimuli responsive, local regional therapy, progress

## Abstract

Cancer seriously threatens human health. Surgery, radiotherapy and chemotherapy are the three pillars of traditional cancer treatment, with targeted therapy and immunotherapy emerging over recent decades. Standard drug regimens are mostly executed via intravenous injection (IV), especially for chemotherapy agents. However, these treatments pose severe risks, including off-target toxic side effects, low drug accumulation and penetration at the tumor site, repeated administration, etc., leading to inadequate treatment and failure to meet patients’ needs. Arising from these challenges, a local regional anticancer strategy has been proposed to enhance therapeutic efficacy and concomitantly reduce systemic toxicity. With the advances in biomaterials and our understanding of the tumor microenvironment, in situ stimulus-responsive hydrogels, also called smart hydrogels, have been extensively investigated for local anticancer therapy due to their injectability, compatibility and responsiveness to various stimuli (pH, enzyme, heat, light, magnetic fields, electric fields etc.). Herein, we focus on the latest progress regarding various stimuli that cause phase transition and drug release from smart hydrogels in local regional anticancer therapy. Additionally, the challenges and future trends of the reviewed in situ smart hydrogels for local drug delivery are summarized and proposed.

## 1. Introduction

Cancer is a global health problem and has become a leading cause of mortality [1,2]. Currently, various treatment options are available to patients with cancer according to different diagnoses and tumor stages, including surgery, chemotherapy and radiotherapy, as well as the newly developed immunotherapy and molecular targeted therapy [1,3]. Despite these treatments now being widely applied in various cancers, some limitations are inevitable, such as the serious side effects associated with treatment, the limited efficacy and damage to normal organs. For instance, the high and non-selective cytotoxicity of chemotherapeutic agents seriously restricts their clinical application, which prevents the clinical use of high doses of drugs and causes damage to various important organs, leading to an unsatisfactory treatment outcome [4,5]. Hence, the design and construction of novel efficient and low-toxic drug delivery systems for cancer therapy have garnered increasing interest and attention.

Local administration of anticancer drugs is characterized by lower drug dose requirements and less toxicity to non-targeted tissues compared with systemic administration, which is expected to achieve greater efficacy and fewer systemic side effects. Various formulations have been investigated to directly distribute drugs to the tumor site, such as gels, suspensions, particles and films [6,7,8,9]. Among these, hydrogels with a three-dimensional (3D) crosslinked network can absorb and retain a large amount of water, and are capable of encapsulating one or more drugs in the crosslinked 3D network [10]. In addition, hydrogels can maintain a moist environment at the application site, and prolong the drug release and avoid it spreading to other healthy areas. Consequently, hydrogels are considered an effective topical drug delivery strategy.

In the context of local administration, the injectability of hydrogel facilitates localized application, avoiding surgical injury. Injectability can be achieved by in situ sol–gel phase transition of the hydrogel composition once it is injected into the tumor site, thus forming drug depots for sustained drug release [11,12]. Smart hydrogels, also called stimulus-responsive hydrogels, have excellent injectability and have gained remarkable attention for their great potential to enhance drug efficiency and reduce systemic adverse effects. Compared with conventional hydrogel systems, in situ smart hydrogel systems exhibit the ability to change their properties, such as phase transition, swelling or shrinking in response to endogenous or exogenous stimuli (e.g., pH, light, temperature, enzymes, electrical fields, magnetic fields, etc.), resulting in injectability and controlled drug release [13,14]. Injectable in situ smart hydrogels are characterized by biocompatibility, negligible cytotoxicity, an outstanding drug loading ratio and controlled drug release, and have been extensively studied for cancer therapy in recent years [15].

**Figure 1:**
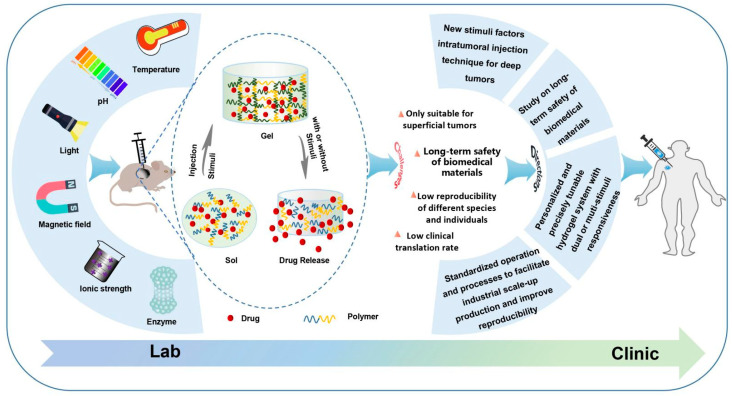
Schematic formation process and drug release of in situ smart hydrogel and challenges and future directions.

The present review aimed to explore the most recent advances in situ smart hydrogels for local antitumor therapy (Figure 1). First, the characteristics and significance of in situ hydrogels for local administration were reviewed. Next, representative smart hydrogels responding to various stimuli were extensively reviewed, including the factors, such as temperature, pH, light, ionic strength, magnetic field, enzymes and electrical fields. In this section, the construction methods and advances of these in situ hydrogels in the last 3 years are summarized and discussed. Finally, the challenges and prospective future trends of injectable in situ smart hydrogel formulations for local antitumor therapy are discussed and proposed.

## 2. Characteristic of In Situ Smart Hydrogels for Local Administration

**Figure 2:**
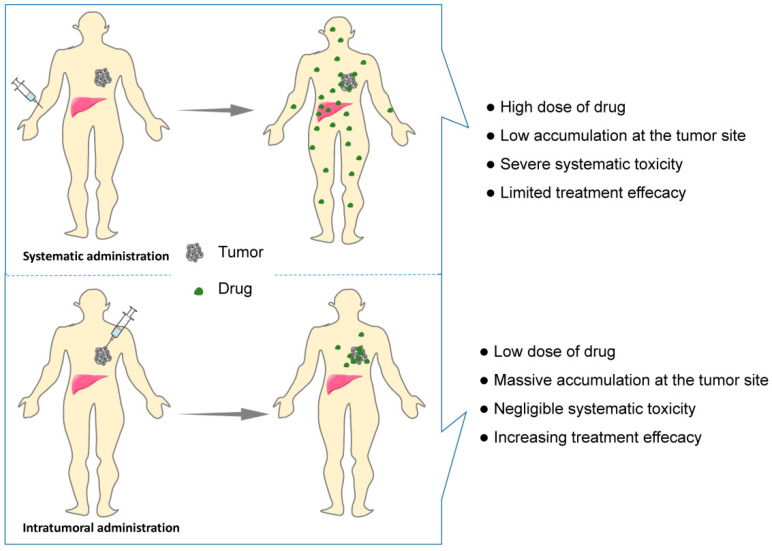
Schematic showing systematic vs. intratumoral administration of anticancer drugs. Comparing with systematic administration, drug biodistribution is localized to the tumor site by intratumoral administration, resulting in lower drug dose requirement and less systematic adverse effect and higher treatment efficacy.

Intravenous drug delivery remains the dominant route of administration for cancer treatment. However, the greatest challenges of intravenous drug delivery, namely poor targeting that induces low tumor accumulation and high systemic toxicities to healthy organs, continue to confound oncologists (Figure 2). In addition, long treatment cycles and repeated administration not only induce low compliance but also increase the economic pressure on patients. Hence, multiple efforts have been made to develop novel antitumor therapy strategies with lower toxicity.

Over the last two decades, ongoing efforts have been made to implement targeted therapies, such as nanotechnology, which have been designed to improve the efficacy and reduce side effects of chemotherapy through passive or active targeting [4,16]. However, systemic treatment by a drug delivery system based on nanotechnology still faces challenges due to burst release, poor bioadhesion, accumulation in non-target tissues, the difficulty of industrial production and clinical transformation [17]. It has been reported that the median accumulation of nanoparticles in the tumor site was only 0.7% of the injected dose, which leads to off-target effects, systemic toxicity and potential long-term nanotoxicity [18,19]. The low tumor accumulation of nanoparticles has also led to reconsideration of the reliability of the enhanced permeability and retention effect (EPR effect), a classic mechanism for passive targeting of nanoparticles to tumors [20].

Local delivery of chemotherapeutic agents can enrich the accumulation of drugs in the injection site and reduce systemic toxicity, allowing low doses to achieve low toxicity and high efficacy (Figure 2). Nevertheless, the intratumoral injection of a drug solution may cause rapid diffusion, leading to a decreasing drug dose within the tumor and toxicity to the healthy tissues [21]. With advancement in biomedical materials, rapid progress in hydrogel-based localized cancer treatment has been witnessed in recent decades. The 3D network structure of hydrogels allows antitumor drugs to be reserved in the hydrogels and to be continuously released from the hydrogels into the tumor site, which limits drug toxicity within a localized area. Compared with aqueous solution of drugs, the in situ hydrogel system, as a novel drug delivery system, has the characteristics of local delivery at the lesion site, prolonged release time and reduced the dosage of drugs [22]. Moreover, the injectability of hydrogels can be achieved by the sol–gel phase transition, which avoids painful surgical implantation and increases the compliance of patients. Smart hydrogels with injectability and stimulus-responsive ability are of great interest in the field of localized anticancer therapy and have become a hot topic. Generally, an ideal in situ smart hydrogel as a localized and continuous drug carrier is required to be characterized by biodegradation, low toxicity, good biocompatibility, suitable gel strength and gelation rate, and a rapid response to internal or external stimuli after injection [23,24]. Consequently, an in situ smart hydrogel drug loading system has great potential to enhance the effectiveness and minimize the adverse effects via intratumoral administration.

## 3. Classification of Smart In Situ Hydrogels

**Figure 3:**
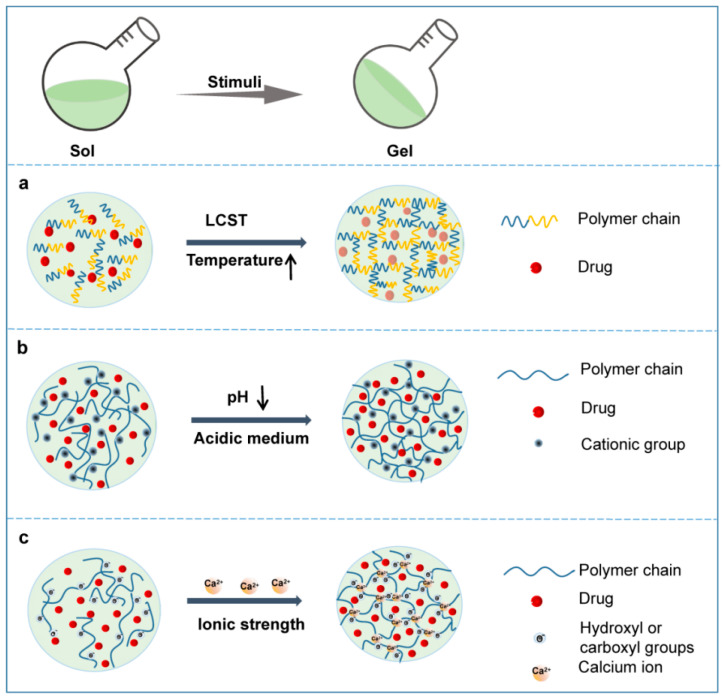
Schematic phase transition of representative hydrogels. (**a**) Temperature responsive hydrogel; (**b**) pH responsive hydrogel; (**c**) Ionic strength responsive hydrogel.

**Figure 4:**
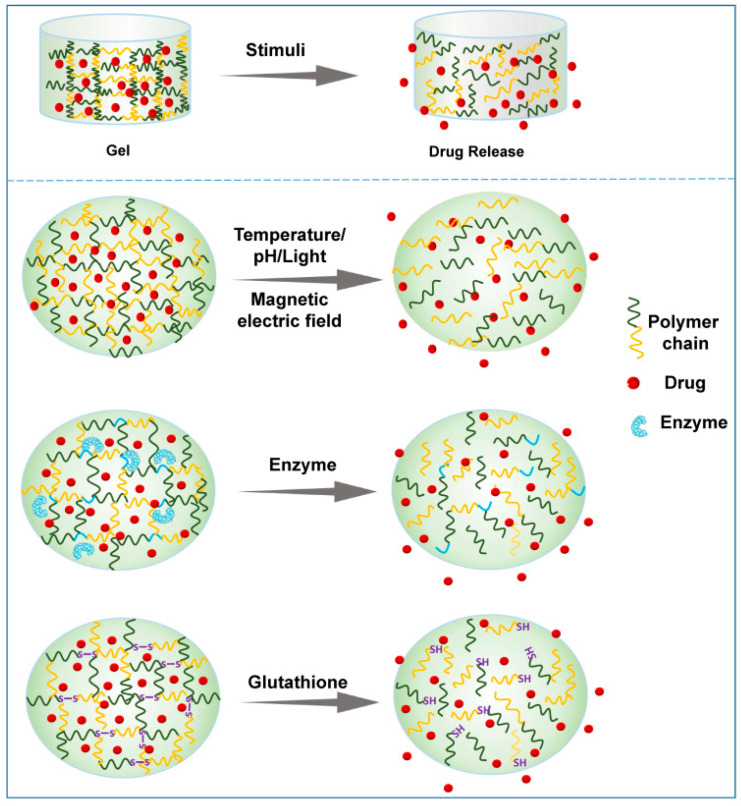
Schematic presentation of drug release from representative smart hydrogels upon various stimuli.

Hydrogels can be classified by various parameters, such as the shape and size, the charge of the hydrogel-forming macromolecules and the mechanism of crosslinking during hydrogel formation [25,26]. The crosslinking process between macromolecules is an important step in creating the 3D structure of the hydrogel. According to the different crosslinking process, hydrogels can be roughly divided into physical hydrogels and chemical hydrogels. Physical hydrogels are formed by physical interactions, such as hydrogen bonding, ionic interactions, hydrophobic interactions, etc. Hydrogels formed through physical processes are reversible and self-healing, with relatively poor mechanical strength [27,28]. The 3D network of chemical hydrogels is generally formed by covalent bonds, and a crosslinking agent is usually required to be added as a trigger of polymerization. Chemical hydrogels have higher mechanical strength but poorer biodegradability compared with physical hydrogels [29]. In this context, considering their ability to change the properties in response to endogenous or exogenous stimuli, smart hydrogels can be divided into various smart hydrogels, such as temperature-, light-, pH-, enzyme- stimulus-responsive hydrogels (Figure 3). In fact, these smart hydrogels can be produced through physical and/or chemical crosslinking. After the formation of drug-loading hydrogels with stimuli, the therapeutic agents can be released into the surrounding environment by diffusion via water-filled pores, implanted matrix, osmotic pumping or matrix erosion of the bulk and surface [30]. Intriguingly, the manner of drug release is well controlled in some kinds of smart hydrogels in response to various stimuli, such as enzyme, glutathione, and magnetic and electrical fields (Figure 4). The ability to react to the stimuli in the environment make smart hydrogels more appropriate candidates for tumor therapy systems.

### 3.1. Temperature Responsiveness

Temperature-responsive hydrogels, which are capable of responding to temperature changes, have been extensively studied among the smart hydrogels [31,32]. As the temperature rises above or drops below a certain point, sol–gel phase transition of the thermoresponsive polymer solution occurs immediately and swelling or shrinking of the volume of the matrix happens simultaneously [10,33]. The temperature at which the phase transition proceeds is named the critical solution temperature (CST) [10]. According to the distinct phase transitions in response to temperature changes, thermoresponsive hydrogels are divided into lower CST (LCST) and upper CST (UCST) hydrogels.

For LSCT hydrogels, the polymer matrices are miscible with water at a temperature lower than the LCST, but the solubility drops dramatically at or beyond the LCST, and then a sol–gel phase transition occurs (Figure 3a) [10]. Hydrophobic interactions are considered as the main force behind the abovementioned phenomenon. Generally, the polymer matrix consists of amphiphilic macromolecules. At a temperature lower than the LCST, the hydrogen bonds between the hydrophilic moieties of the polymer chains and water are formed and strengthened to achieve high solubility (Figure 3a). When the temperature rises, the hydrogen bonds weaken and the hydrophobic interactions between the hydrophobic moieties become stronger, resulting in a dramatic decrease in the solubility of the polymer matrix, the onset of phase transition and the formation of hydrogels [10].

In contrast to LCST systems, the phase transfer in UCST systems is reversed. Briefly, as the temperature rises, the solubility of the polymer matrix increases, and the system exhibits a gel-sol transition at the USCT. Notably, the phase transition temperature of most USCT systems is usually below 25 °C [34]. Hydrogels for biomedical applications require a CST near physiological temperature, namely 30–37 °C [35]. Therefore, hydrogels with an USCT are almost useless in biomedical applications, so here, we mainly focus on thermoresponsive hydrogels with an appropriate LCST rather than USCT. The viscosity of the hydrogel matrix increases abruptly once the temperature is higher than the LCST. As the sol–gel phase transition occurs, the drugs in the system are reserved in the formed hydrogel concurrently and then continuously released from the hydrogel around the tumor site (Figure 4). Moreover, hyperthermia facilitates the cellular uptake of the drugs diffused from the hydrogel because the cytomembrane permeability increases with increasing temperature.

Thermoresponsive hydrogels are based on natural (e.g., chitosan) or synthetic (e.g., poloxamers and polyesters) macromolecules. We consulted the most recent literature on studies of temperature-responsive hydrogels for intratumoral drug delivery in tumor treatment. The polymer materials, therapeutic agents, as well as in vitro and in vivo cancer models of some of the latest reported thermoresponsive hydrogels are summarized in Table 1.

Chitosan (CS), a cationic polysaccharide, produces thermoresponsive hydrogels through interactions with anionic molecules such as β-glycerol phosphate disodium salt (β-GP) at physiological temperature [36]. For instance, Mei et al. designed a novel temperature-responsive hydrogel (CS/GP/HA) constructed from CS, β-GP and hyaluronic acid (HA) [37]. The addition of HA improved the mechanical strength and shortened the gelation time of traditional CS/GP hydrogels. The gelation temperature of traditional CS/GP hydrogels is near body temperature (37 °C) with a gelation time of about 150 s. However, the novel CS/GP/HA hydrogel gelated at a temperature lower than 20 °C with a much shorter gelation time. In another study, Zhang et al. selected silk sericin, CS and β-GP as the matrix materials of a thermosensitive hydrogel loading tegafur (TF)-protoporphyrin IX (PpIX) heterodimers [38]. After intratumoral injection, the sol system transformed into an immovable drug reservoir rapidly at body temperature. The results showed that this thermosensitive hydrogel exhibited long-term drug release and laser-irradiation-responsiveness, achieving on-demand drug administration. The thermosensitive hydrogel with dual drug loading resulted in extremely high synergistic tumor suppression and a significant decrease in the systemic toxicity of the chemotherapy drug. Although CS/b-GP hydrogels possesses significant advantages, namely, thermoresponsive ability, non-toxicity and high biocompatibility, the degradation by erosion and cracking of CS/b-GP hydrogels in vivo impairs their mechanical properties and controlled drug release. Thus, various synthetic polymer biomaterials have been investigated to develop in situ formation of thermoresponsive hydrogels with better mechanical strength.

Poloxamers, triblock copolymers of PEO-b-PPO-b-PEO, are classic thermosensitive and biodegradable synthetic polymers. The LCST of poloxamers is close to physiological temperature when the molecular weight, composition and concentration are adequate, which is important in biomedical applications. Micelle formation theory could be used to explain the gelation mechanism due to the amphiphilicity of poloxamers [10,39]. Thermoresponsive hydrogels based on poloxamers have been widely studied for intratumoral drug delivery. Gao et al. designed an in situ injectable thermosensitive hydrogel system based on pluronic F127 (PF127) for the simultaneous delivery of norcantharidin nanoparticles and doxorubicin (DOX) [40]. The prepared dual drug-loaded hydrogel system exhibited good thermosensitive properties near body temperature as well as controlled drug release. In another study, Li et al. used a mixture of 20 wt% F127 and 6 wt% F68 as a hydrogel matrix material to prepare a thermosensitive hydrogel (BPNSs/DTX-M-hydrogel) simultaneously carrying black phosphorus nanosheets (BPNSs) and docetaxel (DTX) for synergistic photodynamic and chemotherapy [41]. The study showed that the sol–gel transition temperature of the BPNSs/DTX-M-hydrogel was 33 °C. After intratumoral injection, the drug-loaded solution transformed to a stationary hydrogel rapidly at body temperature, and worked as a sustained and localized depot. An in vivo study showed that synergistic therapy with the BPNSs/DTX-M-hydrogel increased antitumor efficacy and reduced the side effects with outstanding biodegradation and biocompatibility.

Although poloxamers are biodegradable and biologically inert, the development of hydrogels made from poloxamers has been limited by the low viscosity and poor mechanical properties. Accordingly, significant efforts have been made to construct thermoresponsive hydrogels based on other synthetic polymers, such as PCLA-PEG-PCLA [42], PLEL [11] and PEG-PLLeu [43]. For example, Zhou et al. synthesized PLEL, an amphiphilic block copolymer, from methoxypolyethylene glycol (mPEG) and d,l-lactide (LA) as a hydrogel matrix by the ring-open polymerization method [44]. Zhou and co-workers prepared erlotinib (ERT)-loaded hollow mesoporous silica nanoparticles (HMSNs) (ERT@HMSNs). Subsequently, the pre-made ERT@HMSN sol was mixed with a prepared cool PLEL sol in a suitable proportion (PLEL copolymer, 15 wt%) to form a homogeneous solution. The gelation temperatures of the ERT@HMSNs PLEL hydrogel systems with 2 mg/mL RET and 6 mg/mL were 35.7 °C and 26.4 °C, respectively, which indicates that this hydrogel system could transform into a physically crosslinked non-flowing gel structure after in situ injection. The long intratumoral and peritumoral drug retention of the ERT@HMSNs PLEL hydrogel provided an impressive balance between antitumor efficacy and systemic adverse effects in a NSCLC xenograft mouse model. Zhang et al. synthesized polyethylene glycol-block-poly-(L-leucine) (PEG-PLLeu) as a thermosensitive hydrogel matrix for simultaneous delivery and consecutive release of regorafenib (REG) and BMS202 [43]. The gelation temperature of the prepared PEG-PLLeu polymer is 4.6 °C, which is suitable for in situ injection. Though the gelation temperature is lower than most thermoresponsive in situ hydrogels, the excellent mechanical properties and biodegradability facilitated the sustained release of the loaded REG and BMS202, and facilitated synergistic suppression of CT26-Luc rectal tumors as well.

**Table 1:** Examples of thermosensitive hydrogels recently developed for localized cancer therapy.

Materials	Therapeutic Agents	Cancer Cell (In Vitro)	Tumor Model (In Vivo)	References
Pluronic F127	Norcantharidin Doxorubicin	HepG2 cells H22 cells	Hepatocellular carcinoma	[40]
Pluronic F127	titanium carbide	4T1 cells	Breast cancer	[45]
PLEL	Erlotinib (ERT),	A549 cells	Lung cancer	[44]
PLEL	indocyanine green R848 CPG ODNs	4T1 cells luciferase labeled 4T1 cells	Breast cancer	[11]
Poloxamer 407	BODIPY-containing, infrared dye-labeled polymeric nanoparticles	GBM-8 cells	Glioblastoma	[46]
Lys-x005f_x005f PCLA	NA	RAW 263.7 cells	NA	[47]
PEG-PLLeu	REG BMS202	CT26-Luc cells	Colorectal cancer	[43]
chitosan and silk sericin	TF-PpIX heterodimers	4T1 cells MCF-7 cells	Breast cancer	[38]
Chitosan and HA	Indocyanine green, imiquimod and cyclophosphamide	A375 cells 4T1 cells	Melanoma Breast cancer	[37]
Methylcellulose	IR820	L929 cells NIH3T3 cells 4T1 cells	Breast cancer	[48]
chitosan	BPNSs CuNPs	A549 cells U87MG cells	Lung cancer hepatocellular carcinoma Glioblastoma	[49]
Pluronic F127	BPNSs DTX	4T1 cells	Breast cancer	[41]
PCLA-PEG-PCLA	curcumin-loaded mPEG-PLA nanopolymersome	C6 tumor cells	Glioma tumor	[42]

Abbreviations: PDLLA-PEG-PDLLA, PLEL: poly(d,l-lactide)-poly(ethylene glycol)-poly(d,l-lactide); Lys-x005f-x0002-PCLA: lysozyme and poly(ε-caprolactone-co-lactide)-b-poly(ethylene glycol)-b-poly(ε-caprolactone-co-lactide) bioconjugate; PEG-PLLeu: poly (ethylene glycol)-block-poly (L-leucine); REG: regorafenib; TF: tegafur; PpIX: protoporphyrin IX; HA: Hyaluronic acid; CTX: Cyclo_x005fphosphamide; ICG: indocyanine green; R837: imiquimod; BPNSs: black phosphate nanosheets; CuNPs: copper nanoparticles; DTX: docetaxel; MP: (methoxy)-polyethylene glycol-b-(polycaprolactone-ran-polylactide) copolymer; St3-shRNA: Stat3-small hairpin RNA; R848: resiquimod; PCLA-PEG-PCLA: poly (ε-caprolactone-co-lactide)-b-poly (ethylene glycol)-b-poly (ε-caprolactone-co-lactide); mPEG-PLA: polyethylene glycol-b-polylactide.

### 3.2. pH Responsiveness

**Figure 5:**
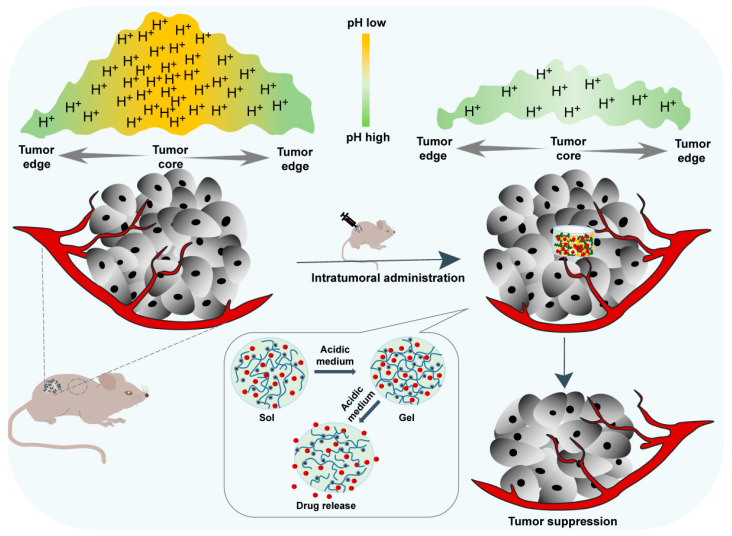
In situ pH-responsive hydrogel alleviates the tumor acidic microenvironment and enhances tumor growth inhibition.

The acidic tumor microenvironment is one of the main characteristics of almost all solid tumors due to the enhanced production of lactate by tumor cells resulting from their high rate of aerobic glycolysis [50]. Such acidification of the extracellular milieu and the increased alkalization of the cytoplasm of the tumor inspired the development of a pH-sensitive local drug delivery system to alleviate acidity within the TME back to the normal pH and suppress tumor growth (Figure 5). Among these, pH-responsive hydrogels have been studied for localized treatment to release cytotoxic drugs exclusively in the tumor site and reduce systematic toxicity.

A pH-responsive hydrogel matrix typically contains a great number of weak acidic or basic groups, including amines, carboxylic acids, imines, etc., which are able to donate or accept protons. When the external pH changes, the properties of these polymers, such as solubility and volume, change due to the donation or acceptance of protons, resulting in phase transition and drug release. For example, after in situ injection, the amine groups of the polymer are positively charged (NH3^+^) by accepting protons because of the acidic external pH of the tumor microenvironment. The chains of the polymer will expand and swell as a result of the electrostatic repulsion between charges (Figure 3b) [51]. The enlarged mesh of swollen hydrogels facilitates drug diffusion through the network. In contrast, acidic groups (-COOH) facilitate the shrinking of the polymer chains in an acidic environment and expansion under alkaline conditions due to the electrostatic interaction. Thus, polymers containing basic groups might be more useful for the construction of hydrogel systems for localized cancer treatment due to the acidic tumor microenvironment. Table 2 lists examples of the latest pH-responsive hydrogels for localized antitumor therapy.

Zhang et al. developed an injectable pH-responsive drug–peptide hydrogel for local chemotherapy [52]. Firstly, methotrexate (MTX), a small-molecule drug with carboxyl groups and 2,3-dimethylmaleic anhydride (DA), a pH-responsive linker, were directly conjugated onto the KKFKFEFEF peptide chain via the amidation reaction, achieving a drug-loaded pH-responsive peptide composite (MTX-KKFKFEFEF(DA)). The impartation of DA to the KKFKFEFEF peptide endowed the peptide with pH-responsive ability. When the environmental pH was 7.4, the MTX-KKFKFEFEF(DA) charged negatively. After intratumoral injection, the negatively charged peptide was activated to be positive due to the acidic tumor microenvironment (pH 6.5), resulting in a sol–gel phase transition. Studies in vitro and in vivo revealed that the MTX-KKFKFEFEF(DA) hydrogel exhibited controlled release and long-term retention in the tumor site, and showed a much better tumor inhibition rate and negligible adverse effects in a breast cancer model as well.

The formation of polyelectrolyte complexes between anionic and cationic polymers is another method of preparing pH-sensitive hydrogels. Many natural polymers (e.g., chitosan, alginate, cellulose and dextran, among others) and synthetic polymers (e.g., polyamines and pyridine derivatives, etc.) have been studied to produce pH-sensitive hydrogels [53,54,55]. Among these, chitosan and its derivatives are among the polymers used most often to produce pH-responsive hydrogels. For instance, Zhan et al. prepared an injectable hydrogel with pH responsiveness and self-healing properties using 4-arm-PEG-benzaldehyde (4armPEGDA) and N-carboxyethyl chitosan (CEC) as a matrix to carry DOX for local treatment of hepatocellular carcinoma [56]. The study results showed that the volume of the prepared hydrogel shrunk at pH 5.6 and expanded at pH 7.4, while the DOX release was fast and substantial at pH 5.6, and slow and sparse at pH 7.4. Accordingly, the acidic tumor microenvironment helped to control the release of the anticancer drug and limit its diffusion to healthy tissues. A strong inhibitory effect on the growth of human hepatocellular carcinoma cells (HepG2) was observed. Importantly, the in vivo antitumor experiment showed that the DOX-loaded pH-responsive hydrogel based on chitosan and 4-arm-PEG could significantly inhibit tumor growth within 5 days, exhibiting better antitumor properties than the other drug-delivery methods, such as tail vein injection.

In addition, some dynamic covalent bonds are pH-responsive groups as well. For instance, polymers abundant in hydrazone bonds are stable at a normal physiological pH (pH 7.4) or under alkaline conditions, whereas the chains will rapidly cleave at a mildly acidic pH. Thus, polymers with such hydrazone bounds will undergo a phase transition as the pH changes. Jia et al. designed a hydrogen bond and hydrazone bond crosslinked pH-responsive hyaluronic acid–collagen (HA–COL) hydrogel, which achieved a sol–gel phase transition quickly in neutral and alkaline environments [57]. In vitro experiments proved that the HA-COL hydrogel gelled quickly in 5 min through the functions of the hydrogen bonds and crosslinking of the hydrazone bonds. Interestingly, a weakly acidic environment may accelerate the degradation of the HA-COL hydrogel and facilitate the release of the loaded drug. Therefore, polymers with an acid-responsive ability appear to hold more potential for in situ drug delivery of antitumor therapy because of the lower pH in the tumor microenvironment than that in healthy tissues.

**Table 2:** Examples of pH-responsive hydrogels recently developed for localized cancer therapy.

Materials	Therapeutic Agents	Cancer Cell (In Vitro)	Tumor Model (In Vivo)	Reference
KKFKFEFEF peptide, 2,3-DA	MTX	4T1 breast cancer cells	Breast cancer	[58]
4armPEG-benzaldehyde, N-carboxyethyl chitosan	DOX	HepG2 cells	Hepatocellular carcinoma	[56]
FOE octapeptide	DOX	4T1 breast cancer cells	Breast cancer	[59]
octa-x005f peptide FEFEFRFK	Paclitaxel	HepG2 cells	H22 tumor	[60]
Dextran phosphate	Prospidine	HEp-2 cells HeLa cells	Cervical epithelial carcinoma	[61]
F-127, Citric acid, 1, 8- Octanediol, PEG-PEI	DOX	A375 cancer cell	Human malignant melanoma	[62]
metal–organic nanoparticles, zinc nitrate	DOX, glucose oxidase	4T1 breast cancer cells	Breast cancer	[63]
PCLA, boronic acid	DOX	HepG2 cells	HepG2 liver cancer	[64]
Chitosan	5-Fluorouracil	HaCaT	Melanoma	[65]

Abbreviations: DA: dimethylmaleic anhydride; PCLA: poly(ε-caprolactone-co-lactide)-b- poly(ethylene glycol)-b-poly(ε-caprolactone-co-lactide; HaCaT: Human keratinocyte cell line; MTX: Methotrexate; PVP: poly-vinylpyrrolidone.

In recent years, peptides, especially self-assembled peptides, have emerged as important structural elements of the hydrogel networks because of their remarkable properties such as versatile functionality, biocompatibility and biodegradability [66,67,68]. Intriguingly, it has been reported that peptide-based hydrogels can change their properties as the pH changes. Li et al. used a pH-responsive ionic-complementary octapeptide FOE as a matrix to develop a DOX-loaded injectable hydrogel, which could self-assemble into a stable hydrogel under neutral conditions and disassemble in the tumor microenvironment [59]. The prepared FOE hydrogel served as a smart drug reservoir delivered via localized injection. The hydrogel exhibited sustained drug release and improved antitumor efficacy in vitro and in vivo.

### 3.3. Light Responsiveness

Light-responsive hydrogels undergo property changes and a phase transition as a consequence of light exposure, including near-infrared radiation (NIR), visible light and UV [69]. Accordingly, there are multiple strategies of preparing photosensitive hydrogels. Incorporation of a photosensitizer, most often photothermal agents, into the hydrogel system is a widely studied method. Photothermal agents generate heat through absorbing and emitting electromagnetic radiation, and then increase the temperature of the system, inducing the phase transition of thermoresponsive hydrogels. Various inorganic and organic photothermal agents have been discovered and studied in recent years, including carbon nanotubes (CNT), graphene (GE) and other carbon nanoparticles, such as gold, silver, porphyrins, cyanines and polydopamine [69,70]. The maximum absorption wavelength of these phototermal agents is mainly in the range of NIR, though this is affected by the materials and surface modifications. Because of the permeability to deep tissue and non-toxicity of NIR light, NIR-induced hydrogels are of great interest. Table 3 lists some examples of recent developed light-responsive hydrogels for local antitumor therapy.

Yang et al. prepared sodium selenite (Se)-directed crosslinked hydrogels based on HA–dopamine loaded with the photothermal agent indocyanine green (ICG) for localized breast cancer therapy [71]. After NIR laser irradiation, the hydrogel-entrapped ICG exhibited excellent photothermal efficacy with significant temperature increases. The photothermal effect of ICG coordinated with the pro-oxidant effect of Se and led to significant tumor inhibition without significant toxic effects. A thermoresponsive self-healing hydrogel containing polydopamine nanoparticles was prepared by dynamic covalent enamine bonds between polyetherimide (PEI) and a four-armed star-shaped copolymer, poly(2-(dimethylamino)ethyl methacrylate-co-2-hydroxyethyl methacrylate), modified with tertbutyl acetoacetate (t-BAA), for localized breast cancer therapy [72]. Due to the photothermal effect of polydopamine, the prepared hydrogel system presented a phase change and volume shrinkage under near-infrared (NIR) irradiation, with an increasing temperature which was above the LCST (40 °C) of the copolymer. Generally, NIR-induced hydrogels are similar to thermoresponsive hydrogels. In addition, some polymers with ionizable functional groups are light-responsive as well. The ionization of polymer chains caused by light radiation results in a change in the volume of the hydrogel matrix, analogous to hydrogel swelling induced by pH changes [33,73]. Given that some photosensitizers show both a photothermal effect and a photodynamic effect, photothermal and/or photodynamic therapy based on in situ hydrogels alone or in combination has also been proposed.

Another typical method of preparing light-responsive hydrogel is based on polymers containing a photoreactive moiety. The photoreactive moiety has the ability to respond to light through photochemical reactions, such as photoisomerization, crosslinking point photopolymerization and photocleavage, which lead to alterations in the hydrogel’s properties, such as the crosslinking density, charging state or hydrophilicity, ultimately inducing phase transition and drug release [55,74]. For example, under UV light exposure, azobenzene and spiropyran undergo reversible trans-to-cis isomerization, while heating or higher wavelength irradiation are able to promote the return to trans isomers [74]. Interestingly, irreversible photocleavage of o-nitrobenzyl and its derivatives occurred under exposure to UV light. In terms of intratumoral drug delivery, photopolymerization is of great importance because it can cause in situ crosslinking and gel formation at the site of administration with UV or visible light exposure [75]. For instance, gelatin methacryloyl (GelMA) was synthetized by functionalization of gelatin with methacryloyl groups, which enabled GelMA photopolymeration by photo-crosslinking with the help of a photo-initiator [76,77]. Through UV or visible light exposure, the photo-initiator in the system absorbs the light energy to generate free radicals, thus leading to polymerization of the GelMA, inducing a sol–gel phase transition. GelMA has been investigated in various fields, including localized drug delivery because of its low immunogenicity, biocompatibility and tunability [78,79,80]. Vigata et al. developed a novel GelMA-based hydrogel loaded with paclitaxel-based abraxane for sustained local delivery of paclitaxel and the prevention of local breast cancer recurrence [79]. In another study, Attia et al. developed a method of in situ photopolymerization of acrylamide hydrogel to carry poorly water-soluble drugs [81]. Luo et al. formulated an injectable multifunctional therapeutic-repair-enabled citrate–iron hydrogel (GPDF) for efficient post-surgical skin cancer treatment by photo-crosslinking gelatin/GelMA in the presence of PCG-dopamine (PCD) and Fe^3+^ ions with UV irradiation [82].

**Table 3:** Examples of light-responsive hydrogels recently developed for localized cancer therapy.

Materials	Therapeutic Agents	Cancer Cell (In Vitro)	Tumor Model (In Vivo)	Reference
Alginate	PpIX-modified Fe3O4 nanoparticles, aPD-L1	4T1 cells	4T1 breast cancer	[83]
Ti3C2, agarose, and protein	HGF, TNF-alpha	DU145 cells	HCT116 colon cancer	[84]
Agarose	WO2.9 nanosheets, nitric oxide (NO) precursor	4T1 cells, C666-1 cells, MCF-7 cells, J774 cells	Breast cancer, hepatoma carcinoma	[85]
Silica colloidal crystal, MAA, HEMA, EGDMA, HMPP	DOX	NIH-3T3 cells, T24 cells	T24 bladder cancer	[86]
Gelatin	BSA/AgNP	B16F10 cells	B16F10 melanoma	[87]
GelMA	Abraxane^®^	MDA-MB-231 cells, MCF-7 cells	NA	[79]

Abbreviations: A549: adenocarcinomic human alveolar basal epithelial cells; PpIX: protoporphyrin IX; aPD-L1: programmed death ligand 1 antibody; HGF: hepatic growth factor; TNF-alpha: tumor necrotic factor-alpha; MAA: Methacrylic acid; HEMA: hydroxyethyl methacrylate; EGDMA: ethyleneglycol dimethacrylate; HMPP: 2-hydroxy-2-methylphenylpropanone; BSA/AgNP: bovine serum albumin (BSA)-coated silver nanoparticles; GelMA: gelatin methacryloyl.

Notably, the photosensitive crosslinkers or initiators in photoresponsive hydrogel systems almost all have varying degrees of toxicity. Additionally, the poor permeability to tissues and potential cancerogenic effect of UV irradiation are another two major obstacles to developing UV-induced photoresponsive hydrogels. The tissue penetration depth of NIR light is deeper than that of UV light. Consequently, a method of upconverting nanoparticles (UCNPs) was developed by scientists to upconvert low-energy photons (e.g., near-infrared, NIR) into high-energy photons (e.g., UV) in order to overcome the poor penetration of UV light and reduce the side effects of UV irradiation on tissues [10,88]. Nonetheless, the tissue penetration depth of NIR light is still limited to superficial tumors, and the application of photosensitive hydrogels to deep tumors is still challenging. Hence, development of biocompatible and non-toxic photosensitizers or photo-initiators are an important direction for photoresponsive hydrogel research.

### 3.4. Magnetic-Field Responsiveness

Magnetic-field-sensitive hydrogels are usually composed of a 3D polymeric network and magnetic nanoparticles, most often iron oxide nanoparticles possessing paramagnetic properties [89,90,91]. These iron oxide nanoparticles can be vibrated through exposure to a magnetic field, resulting in a dramatic increase in the local temperature. Hence, magnetic-field-sensitive hydrogel systems generally combine thermosensitive properties with magnetic-field-responsive ability, in which the temperature increase induced by the magnetic field promotes sol–gel transition and drug release, thus facilitating the synergic efficacy of thermal and chemotherapeutic cytotoxicity. Yan et al. developed a multifunctional in situ formed magnetic hydrogel (NDP-FG) system constructed from a triblock polymer matrix of poly-((N-isopropylacrylamide-co-dopamine)-b-poly(ethylene-glycol)-b-poly (N-isopropylacrylamide-co-dopamine)) (poly-(NIPAM-co-DOPA)-PEG-poly (NIPAM-co-DOPA), NDP) and reduced graphene oxide nanosheets decorated with iron oxide nanoparticles (Fe3O4@rGO, FG) [92]. The temperature of the NDP-FG increased from 20 °C to 49 °C within 10 min after the NDP-FG aqueous dispersion was exposed to the alternating magnetic field, achieving a rapid sol−gel transition. The results indicated that magnetic FG nanosheets contributed to the effective magnetic hyperthermia of the NDP-FG hydrogel, which was demonstrated to be sufficient to kill hepatic cancer cells with negligible side effects on normal cells, leading to highly effective intraoperative hemostasis, and could prevent tumor recurrence. Two main advantages of magnetic-field-sensitive hydrogels are their excellent permeability to deep organs and low invasiveness, which have attracted special attention [34]. However, the results of studies on these treatment strategies combining thermal and magnetic principles were not good enough [93]. The application of magnetic nanoparticles in a hydrogel for localized treatment remains novel, and more research to explore its efficacy or toxic effects is required.

### 3.5. Ionic Strength Responsiveness

Ionic strength-sensitive in situ hydrogels undergo sol–gel phase transition via conformational changes in response to cations such as K^+^, Na^+^ and Ca^2+^ at the administration site. These hydrogels usually consist of ionizable and zwitterionic polymers such as alginate, deacetylated gellan gum, carboxymethyl dextran, polyacrylic acid and carboxybetaine derivatives [24]. Ionic-strength-responsive hydrogels based on these polymers, as a platform for intratumoral administration of cancer therapy, have attracted much attention in recent years due to their biocompatibility, biodegradability and simple fabrication process. Alginate is a typical material used in this kind of hydrogel, which contains abundant hydroxyl and carboxyl groups. When a certain solution of alginate is introduced to solutions containing monovalent (e.g., K^+^) or, more usually, divalent ions (e.g., Ca^2+^), the ions enter the discrete alginate and crosslink with the alginate polymer, achieving a rapid phase transition from a sol to a gel.

Liu’s group prepared in situ hydrogels based on alginate for cancer treatment (Figure 3c) [94,95]. A sodium alginate hydrogel containing catalase (Cat) labeled with the therapeutic 131I radioisotope, the immune adjuvant oligodeoxynucleotides CpG and a checkpoint inhibitor was formulated for localized radioisotope immunotherapy [94]. An in vivo gelation behavior experiment demonstrated the alginate concentration-dependent gel formation process and rapid transformation into gels in the presence of Ca^2+^ when the sodium alginate solution was 5 mg/mL or above 5 mg/mL. Long-term relief of tumor hypoxia and complete tumor elimination at low radioactivity doses were proved by in vivo antitumor experiments on various tumor models. In another study, Liu et al. reported several therapeutic “cocktail” formulations based on an alginate hydrogel by mixing immunogenic cell death (ICD)-inducing chemotherapeutics and immune adjuvants together for localized chemoimmunotherapy [95]. The results showed that in situ gelation of alginate was able to lead to local retention and sustained release of the therapeutics to reduce systemic toxicity. They are now aiming to push this chemoimmunotherapeutic hydrogel cocktail formulation into clinical trials in 2 to 3 years because of the excellent antitumor effects and simple formulation process.

### 3.6. Enzyme Responsiveness

Enzymes are substances with specific catalytic activity and play an important role in almost all metabolic processes in the human lifecycle. The type, activity and quantity of enzymes are different in the physiological or pathophysiological conditions of different tissues and organs. Enzymes, as signals to modulate metabolism, nutrition and energy conversion, are essential during tumorigenesis and tumor development. Importantly, the level of several enzymes in tumors significantly differs from that in normal tissues [96]. Thus, drug delivery platforms based on the enzyme-responsive strategy have been proposed and investigated for years [97,98]. In an enzyme-responsive in situ hydrogel system, the matrix must contain a chemical moiety which needs to be an enzyme-specific substrate or a substrate mimicking and accessible to the enzymatic active center. The enzymatic reaction occurs after injection into the tumor site, and the biomaterial properties subsequently change, which leads to enzyme-mediated crosslinking or cleavage, inducing gelation or drug release (Figure 4) [99].

Matrix metalloproteinases (MMP) have been found to be overexpressed in a variety of tumors, and are among the factors most widely used to activate smart drug carriers [100]. Li et al. designed a MMP-responsive in situ hydrogel loaded with DOX named NDHM for local chemotherapy of oral squamous cell carcinoma (OSCC) [101]. HA was crosslinked with a matrix metalloproteinase-2 (MMP-2)-responsive peptide (GCRDGPQGIWGQDRCG) through a Michael’s addition reaction to achieve in situ formation of a hydrogel matrix, and then DOX-encapsulated micelles were loaded into the hydrogel. After direct injection of the mixed solution into the tumor site, the hydrogel loaded with DOX was formed, and then the formed NDHM hydrogel continuously degraded to release the loaded DOX because of the overexpression of MMP-2 in OSCC. An in vivo antitumor study demonstrated that tumor growth was significantly inhibited by NDHM without evident toxicity. In another study, Tang and coworkers formulated a cytarabine HA-tyramine hydrogel (Ara-HA-Tyr) that incorporated cytarabine, which was formed through the oxidative coupling of tyramines with H_2_O_2_ and horseradish peroxidase (HRP) [102]. An in vivo study showed that the combination of Ara-HA-Tyr and radiotherapy significantly inhibited lung tumor growth and prolonged the survival of tumor-bearing mice in the Lewis lung cancer xenograft model. Notably, the high substrate specificity with regioselectivity and stereoselectivity are important characteristics of such enzyme-sensitive in situ hydrogels. Thus, it is necessary to further explore the specificity of enzymes to their target substrates and to distinguish the distribution of enzymes in different tumor tissues.

### 3.7. Electricity Responsiveness

Electricity-sensitive hydrogels can undergo property changes including swelling, deswelling, bending or erosion in response to electric field exposure [103]. Such electricity-responsive hydrogels are usually based on electrically conductive polymers, inorganic conductive nanomaterials and polyelectrolytes [104]. Accordingly, multiple strategies have been used to design electricity-sensitive hydrogels. Electrically conductive polymers, such as polyaniline (PANI) and polyacrylamide, are also known as ionic electrosensitive polymers. These polymers can change their redox state upon exposure to electrical signals, causing a change in the charge distribution and conductivity. As a consequence, hydrogels composed of such polymers will undergo an alteration in their shape, volume and drug release. Another strategy used to prepare electricity-sensitive hydrogels is the introduction of conductive inorganic nanomaterials, such as carbon nanotubes, graphene and their derivatives, which not only improve the mechanic properties and electrical conductivity of the hydrogels, but also facilitate the drug release under electric field stimuli.

Qu et al. designed a biocompatible biodegradable conductive hydrogel with the ability of precise “on–off” drug release in response external electrical stimuli [105]. An electroactive aniline trimer with hexamethylene diisocyanate was used as the crosslinker and mixed with dextran to form a hydrogel. The hydrogel exhibited the desired conductivity and electrical responsiveness, with a controllable swelling ratio. In this conductive dextran-based hydrogel system, drug release could be modified intelligently by altering the voltage [105]. In another study, Yao et al. designed a novel self-driven triboelectric nanogenerator (TENG)-stimulated catalytic (TENG-Cat) system inspired by the electrostatic preorganization effect in natural enzymes [106]. The TENG-Cat system mainly consisted of three core elements: a human self-driven TENG as the electric field stimulator, a nanozyme and a conductive hydrogel made of poly(2,3-dihydrothieno-1,4-dioxin):poly(styrene sulfonate), which could be injected into the tumor tissue to provide electric pulses, generate ROS and increase local accumulation of the loading nanozyme. Notably, the self-driven wearable TENG had high biosafety, could generate electric pulses under motion and exhibited flexible electric field modulation ability. Additionally, the favorable catalytic activity of the nanoenzyme loaded in the hydrogel was achieved by modulating the electric field. An in vivo study on highly malignant 4T1 breast carcinoma-bearing mice showed that the tumor was significantly suppressed by the fabricated TENG-Cat system. The excellent antitumor efficacy of the smart electric stimuli responsive system sheds light on a new therapeutic mode for self-driven at-home local antitumor therapy.

A low current can induce charged substances in the hydrogel composed of polyelectrolytes to move, inducing a change in the volume of the hydrogel and drug release, which is especially suited for drug delivery across the skin in melanoma therapy. Interestingly, some polyelectrolytes can self-polymerize to form in situ hydrogels by electrostatic interactions in the absence of electric fields. Park et al. proposed a novel strategy based on electrostatic interactions using cationic chitosan and anionic HA to prepare a Substance P (SP) analog (SP1)-loaded injectable hydrogel system [107]. The separated cationic and anionic solution quickly formed a hydrogel in situ after intratumoral injection by a dual-barrel syringe, which is a typical example of a hydrogel that responds to electrostatic interaction.

### 3.8. Other Responsiveness

Apart from the abovementioned smart hydrogels, other stimulus-responsive in situ hydrogels have been investigated for local regional cancer treatment as well. The in situ hydrogel matrix undergoes physical or chemical changes in response to stimuli such as glutathione [108], and antigen [109,110], thus leading to swelling, shrinking, deforming or decomposition. The glutathione-responsive hydrogel is a promising platform for localized antitumor therapy because of excessive intracellular glutathione expression. In such platforms, the basic principle is the inclusion of glutathione-reactive groups (e.g., disulfide), which can be cleaved via acceptance of the electrons from the thiol groups in glutathione [111]. For instance, Li et al. designed and synthesized a novel glutathione (GSH) and pH dual-responsive hydrogel. Alginate was conjugated with glutamic acid–cysteine dendrimer (Glu-Cys-SA) via the click reaction and was then crosslinked with polyethylene glycol (PEG) via hydrogen bonds to form a hydrogel with a 3D network structure [112]. The inner disulfide bonds of the dendrimer were designed to respond to the GSH in the tumor, inducing disulfide cleavage and resulting in the deconstruction of the network structures and the gradual release of the loaded DOX, which was demonstrated in an in vitro experiment. An in vitro cytotoxicity study on HepG-2 cells proved that the prepared GSH-responsive DOX-loaded hydrogel showed significant antitumor effects and no cytotoxicity against normal cells.

In addition, dual and multiple responsive hydrogels for localized antitumor therapy have received more attention than single responsive hydrogels in recent years with the increasing demand for precision therapy. Given the complex tumor microenvironment, these hydrogels are designed to respond to two or more of the abovementioned stimuli for more precise control over the responsive behavior and improvement in the overall performance and applicability of localized drug delivery. Yuan et al. constructed a hydrogel (PNAGA-DMP-Fe3O4@GO) with multiple responsiveness to NIR, light and/or enzymes with thermosensitive poly(N-acryloyl glycinamide) (PNAGA) loaded with doxorubicin (DOX) and polyester (PE) capped mesoporous silica nanocarriers (MSNs) as well as Fe_3_O_4_ nanoparticle (Fe_3_O_4_ NP)-grafted graphene oxide (GO) [113]. This multi-stimulus–responsive hydrogel not only exhibited gel–sol transition by heating after in situ injection, but performed controllable release of DOX and MSNs. This hydrogel combined easily controllable chemotherapy and hyperthermia and eliminated more than 90% of the tumor cells effectively in both in vitro and in vivo studies, showing effective and accurate tumor treatment with negligible side effects on healthy tissue. In another study, Gou et al. designed a muti-stimulus–responsive hydrogel carrying DOX and Cy7 (DOX/Cy7-hydrogel) based on silk fibroin (SF), an FDA-approved natural polymer, inspired by the multi-responsive capacities (hyperthermia, NIR light, pH, reactive oxygen species (ROS) and GSH) of the previous reported SF nanoparticles [114]. The resulting DOX/Cy7 hydrogel exhibited excellent multi-stimulus responses and underwent a reversible thixotropic sol–gel transition after local injection into the tumor site. Additionally, the study results proved the enhanced on-demand drug release profiles of the hydrogel in response to endogenous (acidity, ROS, GSH) and extrogenous (NIR irradiation) stimuli. Strikingly, the in vivo antitumor study demonstrated that this DOX/Cy7-hydrogel was a strong synergistic chemo/photothermal/photodynamic therapeutic strategy, which could eliminate almost the entire tumor mass, significantly prolonging the survival time of tumor-bearing mice over 60 days without detectable adverse effects.

Despite these reported multi-stimulus–responsive in situ hydrogels exhibiting excellent antitumor effects and negligible side effects, how to achieve individualized and precise treatment and ensure the reproducibility of the therapeutic effects remain a hard nut to crack. Multi-stimulus–responsive hydrogels are often connected with complicated design and fabrication procedures, which are very common obstacles for novel drug delivery systems being applied in the clinic. Accordingly, how to balance the complex design of in situ smart hydrogels with simple scaled-up industrial production is a very challenging direction for the future.

## 4. Summary and Outlook

The present review mainly provides an overview of injectable smart hydrogels responding to various stimuli, including temperature, pH, light, magnetic fields, enzymes, electric field, etc. These hydrogel composites change their properties, and exhibit phase transition and controlled drug release because of the special physical and/or chemical properties of the polymer matrix. Local-regional therapy via in situ smart hydrogel systems has been demonstrated by a large number of studies to improve the therapeutical outcomes with fewer adverse effects. Additionally, hydrogels with dual or multiply responsiveness, usually constructed as a combination therapy strategy, have also been proven to further enhance treatment outcomes and weaken toxicity. Hence, in situ smart hydrogels for local administration have been considered as a novel and promising strategy for anticancer in recent decades.

Although the recent decades have witnessed hundreds of successful examples of injectable hydrogel applications for local-regional therapy, challenges in the development of these hydrogel platforms are still present. Several obstacles that need to be addressed soon and potential development prospects are listed below:

- Most localized therapy strategies of in situ hydrogels are mainly limited to superficial tumors and are unavailable for distant lesions or metastases or deep tumors because external stimuli such as light are unable to penetrate deep tissues. The intratumoral injection technique for deep tumors is also a problem for local administration. Optimistically, with the aid of modern imaging techniques such as ultrasound, CT guidance or laparoscopy, safe and precise injections can be achieved in some deep solid tumors.
- Smart hydrogels have been advanced in recent decades with the development of biomaterials. Although the short-term biocompatibility and safety of most new synthesized biomedical materials have been assessed, the long-term toxicity remains inconclusive and requires further investigation and clarification. Therefore, it can be predicted that the safety, especially the long-term safety of biomedical materials, will become an essential research aspect in the future.
- The responsiveness of hydrogels to the stimuli in human tumor tissues might be different from that observed in the laboratory. Even in vitro experiments under pathophysiological conditions sometimes might not be the same as in vivo studies. Moreover, the in vivo reproducibility of the response of smart hydrogels can be influenced by individuals at different cancer stages. Thus, designing personalized and precisely tunable hydrogel systems with dual-stimulus or muti-stimulus responses to meet different patients’ needs is an important and promising direction. Notably, smart hydrogels that are sensitive to biological factors such as enzymes or antibodies have great potential for conduct precise and individual anticancer therapy in combination with other stimuli, which allows precise targeted delivery of the loaded drug.
- The clinical translation of such in situ hydrogel systems still remains difficult. Numerous studies have stopped at the stage of preclinical studies, while a few in situ smart hydrogels have entered clinical trials. This might be ascribed to the complex synthetic routes of some polymers and the complicated preparation process of drug-loaded hydrogels, resulting in difficulty in industrial-scale production. Accordingly, the design of simple and single-step preparations of such hydrogels is another promising direction. Moreover, standardized operation and testing processes are indispensable for realizing industrial production and ensuring their reproducibility.

Generally, the design and development of injectable in situ smart hydrogels have received increasing attention and represent an attractive research direction in the field of local regional cancer therapy. In contrast to traditional or commonly used therapeutic strategies, smart hydrogel systems exhibit specific characteristics: injectability, in situ phase transition and controlled drug release in response to various stimuli. If we take all these together, in the foreseeable future, it is reasonable to believe that a growing number of these hydrogels will enter clinical trials and application for cancer therapy in the future.

## Data Availability

Data sharing not applicable.
